# Identification of gene mutations in patients with primary periodic paralysis using targeted next-generation sequencing

**DOI:** 10.1186/s12883-019-1322-6

**Published:** 2019-05-08

**Authors:** Sushan Luo, Minjie Xu, Jian Sun, Kai Qiao, Jie Song, Shuang Cai, Wenhua Zhu, Lei Zhou, Jianying Xi, Jiahong Lu, Xiaohua Ni, Tonghai Dou, Chongbo Zhao

**Affiliations:** 1 Department of Neurology, Huashan Hospital, Fudan University, Shanghai, 200040 China; 2 Key Laboratory of Contraceptives and Devices, Shanghai Institute of Planned Parenthood Research, institute of Reproduction and development, Fudan University, Shanghai, 200032 China; 3 Department of clinical electrophysiology, Institute of Neurology, Huashan Hospital, Fudan University, Shanghai, 200040 China; 4 State Key Laboratory of Genetic Engineering, Department of Microbiology and Microbial Engineering, School of Life Sciences, Fudan University, 200433, Shanghai, China; 5 Department of Neurology, Jing’an District Center Hospital of Shanghai, Shanghai, 200040 China

**Keywords:** Primary periodic paralysis, Targeted next-generation sequencing, Gene panel, Gene mutation distribution, Calcium homeostasis.

## Abstract

**Background:**

Primary periodic paralysis is characterized by recurrent quadriplegia typically associated with abnormal serum potassium levels. The molecular diagnosis of primary PP previously based on Sanger sequencing of hot spots or exon-by-exon screening of the reported genes.

**Methods:**

We developed a gene panel that includes 10 ion channel-related genes and 245 muscular dystrophy- and myopathy-related genes and used this panel to diagnose 60 patients with primary periodic paralysis and identify the disease-causing or risk-associated gene mutations.

**Results:**

Mutations of 5 genes were discovered in 39 patients (65.0%). SCN4A, KCNJ2 and CACNA1S variants accounted for 92.5% of the patients with a genetic diagnosis.

**Conclusions:**

Targeted next-generation sequencing offers a cost-effective approach to expand the genotypes of primary periodic paralysis. A clearer genetic profile enables the prevention of paralysis attacks, avoidance of triggers and the monitoring of complications.

**Electronic supplementary material:**

The online version of this article (10.1186/s12883-019-1322-6) contains supplementary material, which is available to authorized users.

## Background

Periodic paralysis (PP) is characterized by episodes of muscle weakness that occur at irregular intervals due to skeletal muscle ion channelopathies. This highly heterogeneous group of muscle diseases can be further divided into primary and secondary disorders. The characteristics of primary PP are that they are genetic disorders, usually presenting before the age of 20, often with more than one affected generation and with the exclusion of other diseases that can alter serum potassium levels. The most common genetic causes of primary PP include genes, such as calcium voltage-gated channel subunit alpha 1S (CACNA1S), sodium channel α subunit (SCN4A) and potassium voltage-gated channel subfamily J member 2 (KCNJ2), that encode voltage-gated channels in muscle membranes that generate or sustain membrane potentials [1]. Furthermore, missense mutations in Ryanodine receptor type 1 (RYR1), which directly couples with a voltage-gated L-type Ca^2+^ channel (dihydropyridine receptor, DHPR) to generate excitation-contraction and promote the rapid and generalized release of calcium within myofibrils, were recently identified in patients with PP [2, 3].

Prior to the development of next-generation sequencing (NGS), the molecular diagnosis of primary PP had been based on Sanger sequencing of hot spots or exon-by-exon screening of the reported genes. This approach is time-consuming and, most importantly, is confined to the known genes and thus is less likely to identify cases with more complicated genetic etiologies. The targeted NGS technique has been employed to establish molecular diagnosis in patients with primary myopathies and muscular dystrophies during the past 5 years [4–6]. Targeted NGS is considered as a cost-effective strategy for muscle disorders with heterogeneous genetic causes and allows us to broaden the phenotypic spectrum of hereditary myopathies based on newly identified mutations.

Here, we described the application of a targeted NGS panel that includes 10 ion channel-related genes and 245 muscular dystrophy and myopathy-related genes to obtain a genetic diagnosis in a cohort of 60 Chinese Han patients with clinically diagnosed primary PP.

## Methods

### Patient ascertainment

**Fig. 1:**
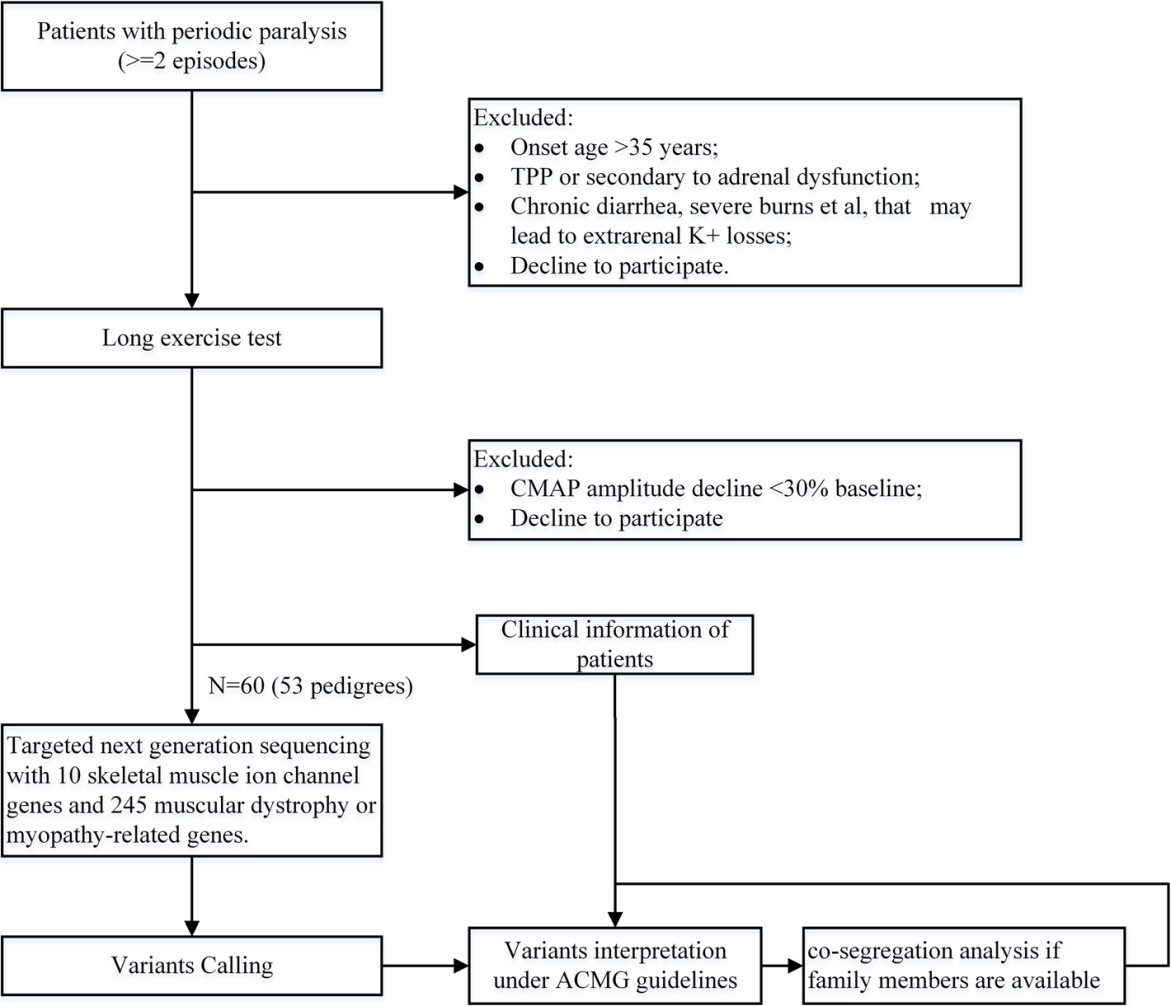
Inclusion and diagnostic strategy for targeted NGS in patients with primary PP. TPP: thyrotoxic periodic paralysis. CMAP: compound muscle action potential

This study was approved under the guidelines of the Institutional Ethics Committee of the Huashan Hospital, and conducted according to the principles in the Declaration of Helsinki. Written Informed consent was obtained from each participant for providing the clinical information and bio-sample for further analysis. For those participants under the age 18, the written consent was signed by their parents. The recruitment of patients with clinically defined primary PP was based on the following criteria: 1) age of onset ≤35 years; 2) recurrent muscle weakness ≥2 times; and 3) a significant decline in the compound muscle action potential (CMAP) amplitude ≥30% of the baseline value in long exercise test (LET) (Fig. 1). The standard procedure of LET was previously described [7]. Serum potassium levels during the attacks either decrease, remain at a normal level (3.5–5.5 mmol/L) or increase. Patients with hyperthyroidism, adrenoid gland dysfunction and renal tubular acidosis are excluded in this study. Taken together, we included a total of 60 patients from 53 pedigrees for further genetic screening with NGS.

### Targeted next-generation sequencing

Genomic DNA was extracted from peripheral blood using the High Pure PCR.

Template Preparation Kit (Roche, Basel, CH) according to the manufacturer’s instructions. The DNA fragments were enriched by performing solution-based hybridization capture, followed by sequencing using an IlluminaMiseq platform (Illumina, San Diego, CA, USA) with the 2 × 300 bp paired-end read module. The hybridization capture procedure was performed using the SureSelect Library Prep Kit (Agilent, Santa Clara, CA, USA). The defined target regions included 5060 regions containing 255 genes known to cause muscle disorders, including ion channelopathies, limb-girdle muscular dystrophies, Duchenne/Becker muscular dystrophies, congenital muscular dystrophies, metabolic myopathies, congenital myopathies and distal myopathies (Additional file 1 Table S1). The oligonucleotides covered all coding exons, UTR regions and intron-exon boundaries, including at least 10 intronic nucleotides. The DNA was first quantified using Qubit 2.0 (Thermo Fisher Scientific, Waltham, MA, USA). In total, 3 μg of genomic DNA were sheared by sonication using a DiangenodeBioruptor® Plus and hybridized using Biotinylated RNA oligonucleotide baits. The captured fragments were removed from the solution using streptavidin-coated magnetic beads (Dynabeads® MyOne™ Streptavidin T1, Thermo Fisher Scientific) and subsequently eluted. The resulting libraries were quantified by qPCR before proceeding to the Illumina Miseq platform.

### Variant analysis and interpretation

After the Miseq sequencing, the raw reads from each sample were sorted according to the index sequences. The adapter sequences were trimmed using cutadapt (http://code.google.com/p/cutadapt/). SolexaQA was used to remove the low-quality bases (< Q20). The clean reads were aligned to the human reference genome (hg19) using the Burrows–Wheeler Aligner (BWA; ver. 0.7.11) [8–10]. After the alignment, the PCR duplicates were removed using the Picard tools (ver. 1.109) Mark Duplicates package. The realignment around the known indel sites and Base Quality Score Recalibration (BQSR) were performed using GATK (ver. 3.3) [11]. GATK HaplotypeCaller was used to call the raw variants. The indels and SNPs were annotated using ANNOVAR [12]. Public databases, including dbSNP138, 1000 Genome project, Exome Sequencing Project, ClinVar and HGMD [13], were used to screen the variants. The functional effect prediction was evaluated using the PolyPhen-2 and SIFT scores [14, 15]. To detect the copy number variant (CNVs), the sequencing depth of each region covered by the probes was calculated according to the alignment files. The ExomeDepth [15] package was also used to identify potential CNVs. Confirmed point mutation samples in the same sequence run served as controls in the CNV analysis. All variants are classified in American College of Medical Genetics and Genomics standards and guidelines [16].

### Variants verification

To further verify the candidate mutations, we performed Sanger sequencing of the DNA samples extracted from the patients and their family members. PCR was performed using GoldStarTaq DNA Polymerase (CWbiotech) according to a standard protocol [17]. The PCR products were sequenced on an ABI3730xl DNA Analyzer (Applied Biosystems). A genotype-phenotype co-segregation analysis was performed if the parents’ blood samples were available. To detect splicing mutations, RNA was extracted from frozen muscle biopsies using RNAiso Plus (Takara), and cDNA was synthetized using a PrimeScriptTMRT Reagent Kit (Takara).

## Results

### Sequence reads and average coverage of the targeted regions

We performed targeted NGS in 60 patients with clinically defined primary periodic paralysis from 53 families to identify 17 known variants and 8 novel mutations in skeletal muscle ion channel genes (Additional file 2: Table.S1). These variants included 2 pathogenic, 16 likely pathogenic and 7 variants with uncertain significance. No CNVs have been found in these cases.

On average, 3625 raw variants were detected per patients, and 3154 high-quality variants remained after filtering. The average sequencing depth was 488.11×, and the average coverage was 99.45%. GC-rich sequences have been shown to reduce the efficiency of the probe capture. Ion channel genes with GC-rich (> 70%) target regions are listed in Additional file 2: Table. S2. Within these regions, a much lower sequencing depth and coverage have been observed.

### Variants with established skeletal muscle ion channel-related genes

**Fig. 2:**
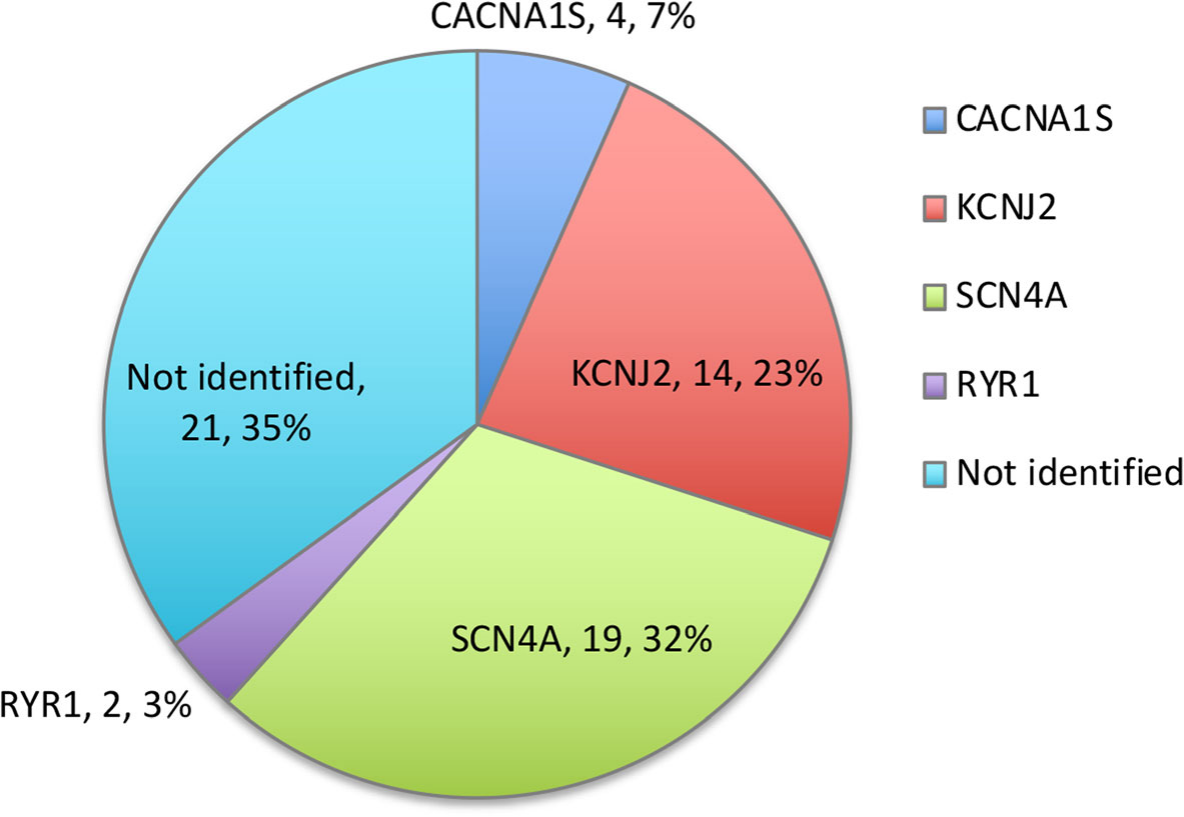
Proportions of different gene variants

**Fig. 3:**
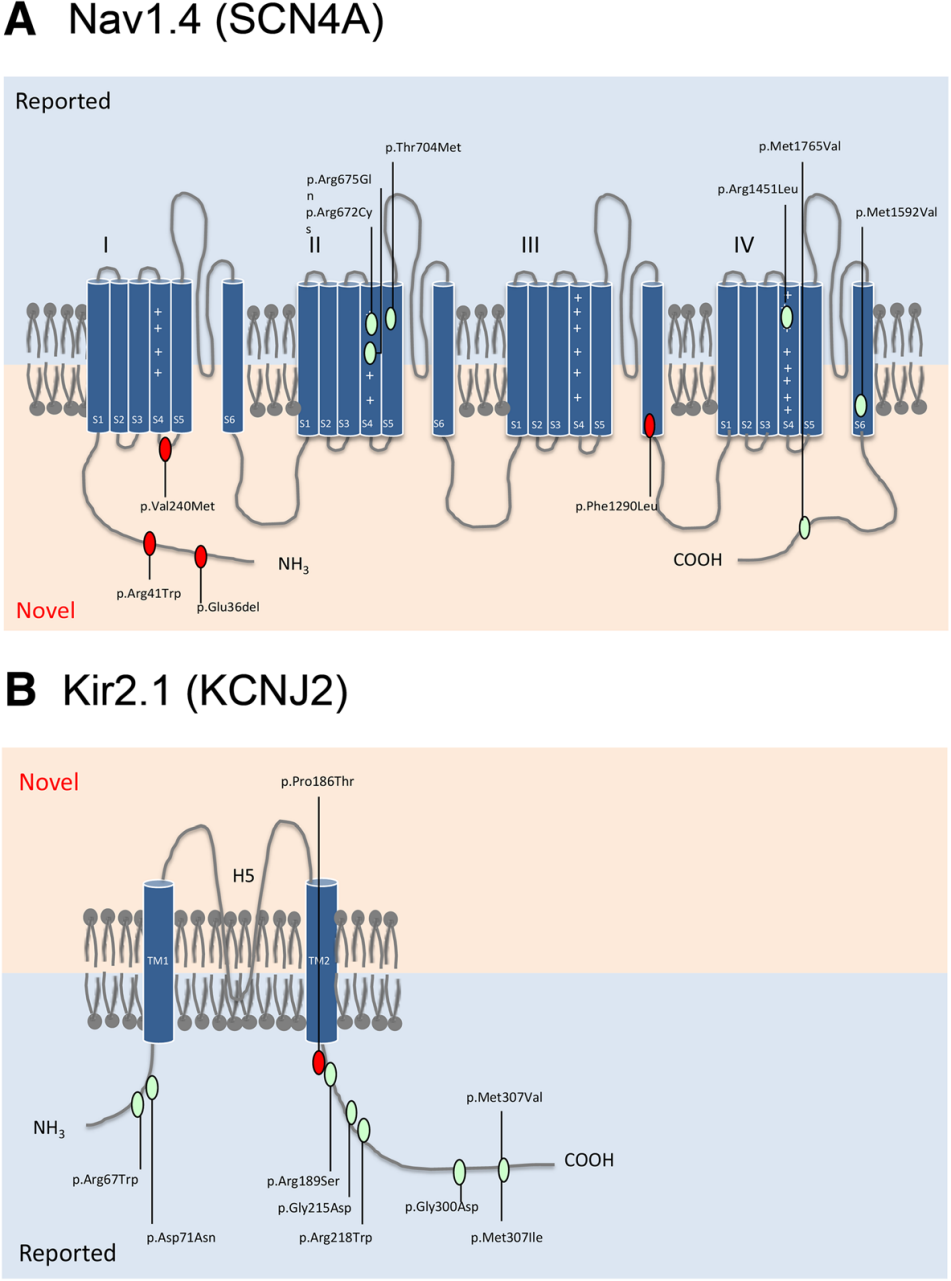
Distribution of SCN4A and KCNJ2 variants in the Nav1.4 (**a**) and Kir2.1 structure (**b**). Red ovals depict novel variants and green ovals are reported variants

Variants of established skeletal muscle ion channel genes, including CACNA1S, SCN4A and KCNJ2, were identified in a total of 37 patients (37/60, 61.67%)(Fig. 2). SCN4A (NM_000334) mutations accounted for the majority in this primary PP cohort (19/39,48.7%). We identified 10 SCN4A variants including 6 reported mutations (c.2014C > T p.Arg672Cys, c.2024G > A p.Arg675Gln, c.2111C > T p.Thr704Met, c.4352G > T p.Arg1451Leu, c.4774A > G p.Met1592Val, and c.5293G > A p.Ala1765Thr) and 4 novel mutations (c.107_109del p.Glu36del, c.121C > T p.Arg41Trp, c.718G > A p.Val240Met, and c.3868 T > C p.Phe1290Leu) (Fig. 3a). Among them, c.2024G > A p.Arg675Gln was identified in 8 patients from 6 families as a potential hotspot. Two variants with uncertain significance (c.107_109del p.Glu36del and c.5293G > A p.Ala1765Thr) are distributed in the N′- terminus and C′-terminusof the α-subunit, which are distant from the 24 α-helical transmembrane segments.

Second most prevalent gene responsible for primary periodic paralysis was KCNJ2 (NM_000891). We identified 8 reported variants (c.199C > T p.Arg67Trp, c.211G > A p.Asp71Asn, c.566G > T p.Arg189Ser, c.644G > A p.Gly215Asp, c.652C > T p.Arg218Trp, c. 899G > A p.Gly300Asp, c.919A > G p.Met307Val, and c.921G > C p.Met307Ile) in a total of 14 patients (Fig. 3b). Moreover, we identified 4 likely pathogenic variants (c.614 T > A p.Phe205Tyr, c.1583G > A p.Arg528His, c.2965G > A p.Glu989Lys and c.3716G > A p.Arg1239His) in CACNA1S gene (NM_000069).

### Clinical features of patients with known skeletal muscle ion channel-related gene mutations

Patients with SCN4A mutations are all male with juvenile-onset hypoPP or normoPP. Noticeably, one case had normal serum potassium level during attacks (3.8 mmol/L, normal, 3.5–5.5 mmol/L), but the muscle weakness got worsened by steroids administration. We identified a reported homozygous SCN4A mutation (c.4352G > T p.Arg1451Leu) in a teenage boy with hypokalemia [18, 19]. The parents are both heterozygous carriers of the same variant but only manifest subclinical myotonia after EMG study.

A total of 14 patients (14/39, 35.9%) with ATS manifested early onset periodic paralysis, variable dysmorphic features and cardiac arrhythmia, except one case whose ECG result is not available. Ventricular arrhythmia including premature ventricular contraction, bigeminy runs, couplets, triplets and bidirectional ventricular tachycardia, was demonstrated in 13 cases (except P16 who did not perform ECG study, Additional file 1: Table.S1). Long QT syndrome was documented in 11 cases.

All 4 cases (4/39,10.2%) with CACNA1S mutations are either autosomal dominant inherited (2 patients) or sporadic periodic paralysis (2 patients) with hypokalemia. Potassium supplement and methazolamide oral administration effectively reduced the episodic frequency.

### Variants in rarely reported skeletal muscle ion channel-related genes

One known variant (c.8290G > A p.Glu2764Lys) and one novel variant (c.12428C > T p.Ala4143Val) were identified in RYR1 gene. The patient harboring heterozygous RYR1 variant p.Glu2764Lys had repeated episodes of generalized normokalemic paralysis after 20 years old. Another patient carrying heterozygous RYR1 variant p.Ala4143Val complained of recurrent muscle weakness in proximal lower limbs with hypokalemia since 24 years old with a frequency of 2–3 times per year. Neither of these cases had proceeding family history of neuromuscular disorders. There is no cognitive, bulbar or respiratory involvement.

## Discussion

In this study, we designed a targeted NGS gene assay comprising 10 ion channel-related genes and 245 muscular dystrophy/myopathy-related genes to screen patients with primary PP who were referred to our diagnostic center. In total, we elucidated genetic contributions in 65.0% (39/60) of the primary periodic paralysis cohort.

Known responsible gene variants (CACNA1S, KCNJ2 and SCN4A) were identified in 61.67% of the whole cohort. So far, this is the first cohort to study the frequencies of known variants in Chinese patients with primary PP, as follows: (1) SCN4A variants are the most common genetic cause; (2) KCNJ2 variants are the second most common; (3) CACNA1S mutation is relatively rare. Among the 37 cases with HypoPP, SCN4A mutation group accounts for 29.73% (11/37), KCNJ2 and CACNA1S account for 10.81% (4/37) respectively. While CACNA1S mutations are the most common in HypoPP patients in USA and European population [20–22], SCN4A accounts for the majority of HypoPP across Chinese individuals. Since most patients recruited in this study are adults, a selection bias may exist. Besides, there are different genetic variations for patients with primary PP across populations. With regard to the hot spot identified in this study, a SCN4A variant (p.Arg675Gln) accounted for the majority of all patients with sodium channelopathies (42.1%, 8/19). The most prevalent sodium channel variants in USA population [21], T704 M and M1592 V, were identified in three Chinese individuals with normokalemic PP.

In this cohort, most variants identified in the Nav1.4 channel were distributed in the S4-S6 transmembrane regions where the voltage sensors and pore formers are located. A Novel variant p.Val240Met was located in the cytoplasmic Domain I S4-S5 loop. The linker help connected the voltage sensing domain S4 and pore-forming domains S5-S6 [23]. At normal conditions, depolarization will cause the positively charged S4 segment to move towards cell surface and this motion is transferred to the pore domain S5–6 via the linker, abruption of which results in a failed conformational change thus lead to delayed opening of the sodium channel. Another novel variant p.Phe1290Leu was located in the N-terminal of Domain III-S6, the pore-forming segment of the channel. The remaining 3 novel variants, i.e.,p.36_37del, p.Arg41Trp and p.Ala1765Thr, are distributed in the N′- terminus and C′-terminus of the α-subunit. Though they are rare variants and might be pathogenic, a detailed cosegragation analysis and functional studies are still required to valid the results.

Nine variants identified in Kir2.1 were located in the C-terminal intracytoplasmic tails. There is no hotspot variant in KCNJ2 identified in this Chinese cohort. These regions are considered to play an important role in maintaining normal function; thus, these mutations likely disrupt the ion channels. Of these mutations, p.Pro186Thr, p.Arg189Ser, p.Gly215Asp and p.Arg218Trp are located at sites in which Kir2.1 and phosphatidylinositol-4,5-bisphosphate (PIP2) combine, which likely abolishes the direct and specific activation of Kir2.1 [24]. Two highly frequent missense variants, i.e.,p.Arg528His and p.Arg1239His in CACNA1S gene were observed in two patients. According to previous reports, patients with these two mutations have a late onset age of 17.2 ± 4.0 and 15.8 ± 8.8 years compared with that of other HypoPP cases [25, 26].

RYR1 gene has originally been reported to be responsible for congenital myopathy and malignant hyperthermia. Two missense mutations of the RYR1 gene were demonstrated in two independent cases with PP in our cohort. The first mutation p.Glu2764Lys was previously found in a patient with malignant hyperthermia [27]. Another mutation p.Ala4143Val was novel with a Polyphen-2score of 0.991 and a SIFT prediction score of 0. This mutation is located in the Malignant hyperthermia domain III (exon 90–104). In a previous study, A homozygous RYR1 mutation (c.8816G > A p.Arg2939Lys) in skeletal muscle was responsible for a 35-year-old male with congenital myopathy as well as a typical periodic paralysis [3]. A discrepancy was demonstrated that the c.8816G > A nucleotide change was heterozygous at the genomic level. However, in our cases with RYR1 heterozygous mutations, it needs further analysis with patient-derived skeletal muscle and cell models to establish the relationship between the RYR1 variants and the phenotype.

**Fig. 4:**
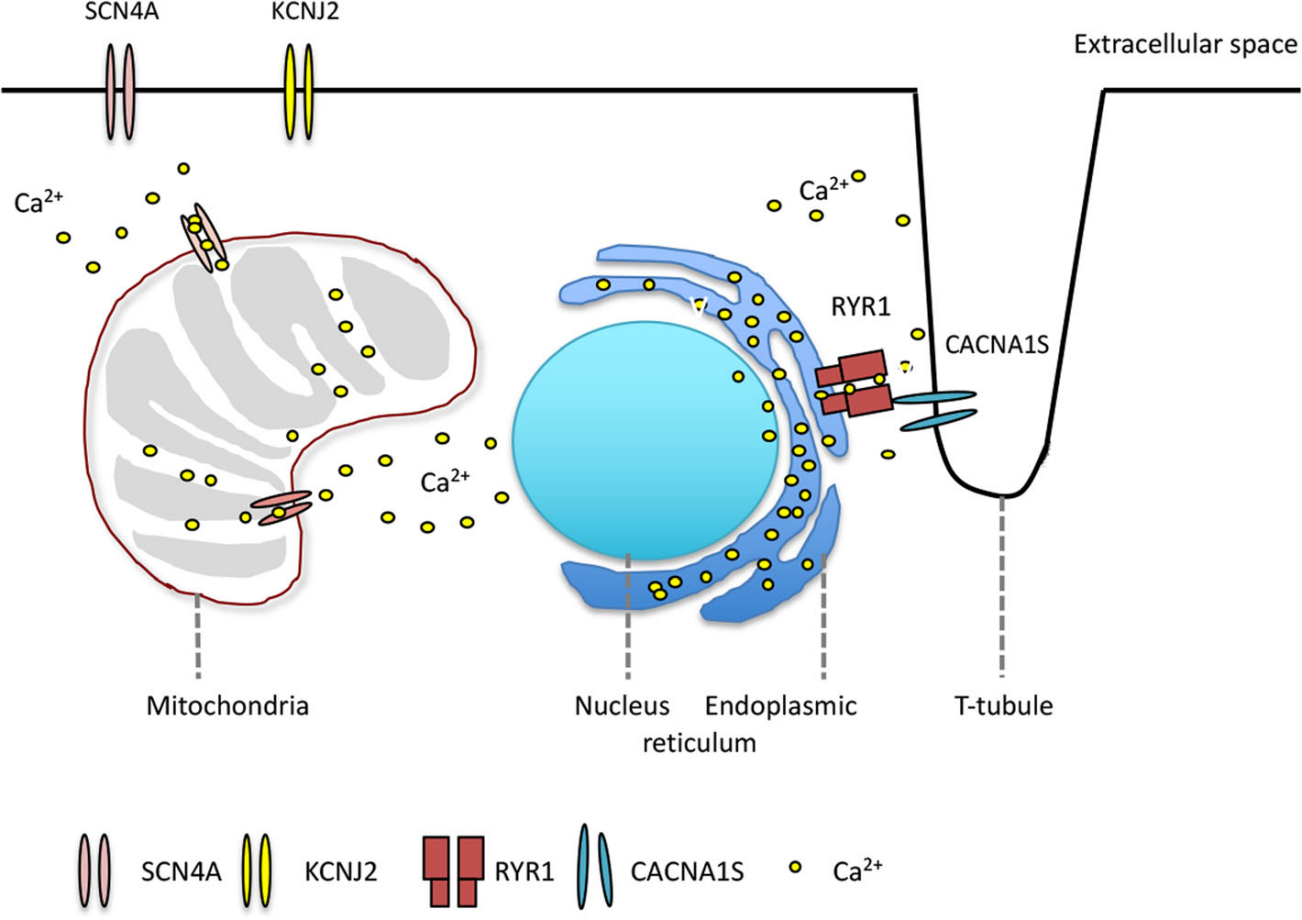
The distribution of identified primary periodic paralysis related proteins

Using this NGS panel, we possibly expanded the spectrum of genotypes associated with PP (Fig. 4). NGS is not a perfect tool but is a method under development for discovering new mutations that may be involved in disease conditions. By including primary myopathy/muscle dystrophy genes in the panel, we increased the diagnostic yield from 61.67% (37/60) to 65.0% (39/60) in screening primary PP patients. But for some rare variants like RYR1 variants, the functional analysis is required for further confirmation of the causality.

Still, we were unable to achieve a definite diagnosis in the remaining 21(35.0%) cases. The relatively low depth and coverage in the GC-rich sequences of certain genes, which are likely causal CNV variations, and unknown genes may contribute to major causes of the panel-negative cases. Hopefully, solving the phenotype positive/genotype negative mystery cases will shed new light regarding novel pathogenic mechanisms underlying the remaining cases of primary PP.

## Conclusions

Using targeted NGS, we achieved a diagnostic success rate of 65% in a cohort of primary PP patients. We expanded the spectrum of genotypes of primary PP and clinical phenotypes of known myopathy-related genes. We’d like to attribute this diagnostic rate to the inclusive strategy of screening for other causal factors of hypokalaemic muscle weakness and performing accurate clinical examinations and history inquiries prior to the interpretation of the NGS results.

## Additional files

Additional file 1: **Table S1.** Clinical features and genetic variants of the patients with primary periodic paralysis in this study. AD: autosomal dominant. AR: autosomal recessive. HypoPP: hypokalemic periodic paralysis. NormoPP: normokalemic periodic paralysis. Novel variants are in red. (DOCX 23 kb)

Additional file 2: **Table S2.** Skeletal muscle ion channel genes with GC-rich (> 70%) regions (DOCX 15 kb)

## Abbreviations

ARVC: Arrhythmogenic right ventricular cardiomyopathy
BQSR: Base quality Score Recalibration
BWA: Burrows-Wheeler Aligner
CMAP: Compound muscle action potential
CNV: Copy number variant
CPEO: Chronic progressive external ophthalmoplegia
DHPR: Dihydropyridine receptor
ER: Endoplasmic reticulum
ExAC: Exome Aggregation Consortium
hg19: Human reference genome 19
HyperPP: Hyperkalemic periodic paralysis
HypoPP: Hypokalemic periodic paralysis
NGS: Next generation sequencing
NormoPP: Normokalemic periodic paralysis
OXPHOS: Oxidative phosphorylation
PIP2: Phosphatidylinositol-4,5-bisphosphate
PP: periodic paralysis
SUNDS: Sudden unexplained nocturnal death syndrome
WES: Whole exome sequencing
WGS: Whole genome sequencing
