# Supplementary material for: Identification of gene mutations in patients with primary periodic paralysis using targeted next-generation sequencing
Source: BMC Neurol. 2019 May 8;19:92. doi: 10.1186/s12883-019-1322-6 (PMC6505267; doi:10.1186/s12883-019-1322-6)
Supplement: Supplementary file 1 — Table S1. Clinical features and genetic variants of the patients with primary periodic paralysis in this study. AD: autosomal dominant. AR: autosomal recessive. HypoPP: hypokalemic periodic paralysis. NormoPP: normokalemic periodic paralysis. Novel variants are in red. (DOCX 23 kb) [file 12883_2019_1322_MOESM1_ESM.docx]

Table 1 Clinical features and genetic variants of the patients with primary periodic paralysis in this study.

| No. | Family No. | Sex | onset age | Inheritance | Periodic paralysis | Gene | cDNA | protein | ACMG |
| --- | --- | --- | --- | --- | --- | --- | --- | --- | --- |
| P1 | 1 | F | 11 | sporadic | HypoP | CACNA1S | c.614T>A | p.Phe205Tyr | Likely pathogenic |
| P2 | 2 | M | 8 | AD | HypoP | CACNA1S | c.1583G>A | p.Arg528His | Likely pathogenic |
| P3 | 3 | M | 25 | sporadic | HypoP | CACNA1S | c.2965G>A | p.Glu989Lys | Likely pathogenic |
| P4 | 4 | F | 11 | AD | HypoP | CACNA1S | c.3716G>A | p.Arg1239His | Likely pathogenic |
| P5 | 5 | M | 7 | AD | NormoP | KCNJ2 | c.199C>T | p.Arg67Trp | Uncertain significance |
| P6 | 6 | M | 12 | sporadic | HypoP | KCNJ2 | c.199C>T | p.Arg67Trp |  |
| P7 | 7 | M | 7 | sporadic | NormoP | KCNJ2 | c.211G>A | p.Asp71Asn | Uncertain significance |
| P8 | 8 | F | 15 | AD | NormoP | KCNJ2 | c.556C>A | p.Pro186Thr | Pathogenic |
| P9 |  | F | 5 | AD | NormoP | KCNJ2 | c.556C>A | p.Pro186Thr |  |
| P10 |  | F | 6 | AD | NormoP | KCNJ2 | c.556C>A | p.Pro186Thr |  |
| P11 | 9 | M | 11 | AD | NormoP | KCNJ2 | c.566G>T | p.Arg189Ser | Likely pathogenic |
| P12 |  | M | 17 | AD | NormoP | KCNJ2 | c.566G>T | p.Arg189Ser |  |
| P13 | 10 | F | 11 | sporadic | HypoP | KCNJ2 | c.644G>A | p.Gly215Asp | Likely pathogenic |
| P14 | 11 | M | 11 | sporadic | NormoP | KCNJ2 | c.652C>T | p.Arg218Trp | Likely pathogenic |
| P15 | 12 | M | 19 | AD | NormoP | KCNJ2 | c.899G>A | p.Gly300Asp | Likely pathogenic |
| P16 | 13 | M | 18 | sporadic | HypoP | KCNJ2 | c.919A>G | p.Met307Val | Uncertain significance |
| P17 | 14 | F | 17 | AD | NormoP | KCNJ2 | c.921G>C | p.Met307Ile | Likely pathogenic |
| P18 |  | F | 1 | AD | HypoP | KCNJ2 | c.921G>C | p.Met307Ile |  |
| P19 | 15 | M | 15 | sporadic | HypoP | SCN4A | c.107_109del | p.Glu36del | Uncertain significance |
| P20 | 16 | M | 20 | sporadic | NormoP | SCN4A | c.121C>T | p.Arg41Trp | Likely pathogenic |
| P21 | 17 | M | 17 | sporadic | HypoP | SCN4A | c.718G>A | p.Val240Met | Likely pathogenic |
| P22 | 18 | M | 16 | sporadic | HypoP | SCN4A | c.2014C>T | p.Arg672Cys | Likely pathogenic |
| P23 | 19 | M | 17 | AD | HypoP | SCN4A | c.2024G>A | p.Arg675Gln | Likely pathogenic |
| P24 | 20 | M | 3 | AD | NormoP | SCN4A | c.2024G>A | p.Arg675Gln |  |
| P25 | 21 | M | 12 | AD | HypoP | SCN4A | c.2024G>A | p.Arg675Gln |  |
| P26 |  | M | 27 | AD | HypoP | SCN4A | c.2024G>A | p.Arg675Gln |  |
| P27 | 22 | M | 20 | AD | HypoP | SCN4A | c.2024G>A | p.Arg675Gln |  |
| P28 | 23 | M | 16 | AD | NormoP | SCN4A | c.2024G>A | p.Arg675Gln |  |
| P29 |  | M | 22 | AD | NormoP | SCN4A | c.2024G>A | p.Arg675Gln |  |
| P30 | 24 | M | 12 | AD | HypoP | SCN4A | c.2024G>A | p.Arg675Gln |  |
| P31 | 25 | M | 5 | sporadic | NormoP | SCN4A | c.2111C>T | p.Thr704Met | Likely pathogenic |
| P32 | 26 | M | 10 | AD | NormoP | SCN4A | c.2111C>T | p.Thr704Met |  |
| P33 | 27 | M | 22 | AD | HypoP | SCN4A | c.3868T>C | p.Phe1290Leu | Pathogenic |
| P34 |  | M | 12 | AD | HypoP | SCN4A | c.3868T>C | p.Phe1290Leu |  |
| P35 | 28 | M | 4 | AR | HypoP | SCN4A | c.4352G>T | p.Arg1451Leu | Likely pathogenic |
| P36 | 29 | M | 3 | sproradic | NormoP | SCN4A | c.4774A>G | p.Met1592Val | Likely pathogenic |
| P37 | 30 | M | 7 | sproradic | NormoP | SCN4A | c.5293G>A | p.Ala1765Thr | Uncertain significance |
| P38 | 31 | M | 20 | sproradic | NormoP | RYR1 | c.8290G>A | p.Glu2764Lys | Uncertain significance |
| P39 | 32 | M | 24 | sproradic | HypoP | RYR1 | c.12428C>T | p.Ala4143Val | Uncertain significance |
| P40 | 33 | M | 18 | sproradic | HypoP |  |  |  |  |
| P41 | 34 | M | 23 | sproradic | HypoP |  |  |  |  |
| P42 | 35 | M | 34 | AD | HypoP |  |  |  |  |
| P43 | 36 | M | 28 | sproadic | NormoP |  |  |  |  |
| P44 | 37 | F | 18 | AD | NormoP |  |  |  |  |
| P45 | 38 | M | 33 | sporadic | NormoP |  |  |  |  |
| P46 | 39 | M | 20 | sporadic | HypoP |  |  |  |  |
| P47 | 40 | M | 25 | sproradic | HypoP |  |  |  |  |
| P48 | 41 | M | 30 | sproradic | HypoP |  |  |  |  |
| P49 | 42 | M | 12 | sproradic | HypoP |  |  |  |  |
| P50 | 43 | M | 12 | sproradic | HypoP |  |  |  |  |
| P51 | 44 | M | 32 | sproradic | HypoP |  |  |  |  |
| P52 | 45 | M | 14 | sproradic | HypoP |  |  |  |  |
| P53 | 46 | M | 15 | sproradic | HypoP |  |  |  |  |
| P54 | 47 | M | 12 | sproradic | NormoP |  |  |  |  |
| P55 | 48 | M | 30 | sproradic | HypoP |  |  |  |  |
| P56 | 49 | M | 13 | sproradic | HypoP |  |  |  |  |
| P57 | 50 | M | 18 | sproradic | HypoP |  |  |  |  |
| P58 | 51 | M | 27 | sproradic | HypoP |  |  |  |  |
| P59 | 52 | M | 12 | sproradic | HypoP |  |  |  |  |
| P60 | 53 | M | 28 | sproradic | HypoP |  |  |  |  |

Tab.1 Clinical features and genetic variants of the patients with primary periodic paralysis in this study. AD: autosomal dominant. AR: autosomal recessive. HypoPP: hypokalemic periodic paralysis. NormoPP: normokalemic periodic paralysis. Novel variants are in red.
