# Supplementary material for: Identification of gene mutations in patients with primary periodic paralysis using targeted next-generation sequencing
Source: BMC Neurol. 2019 May 8;19:92. doi: 10.1186/s12883-019-1322-6 (PMC6505267; doi:10.1186/s12883-019-1322-6)
Supplement: Supplementary file 2 — Table S2. Skeletal muscle ion channel genes with GC-rich (> 70%) regions (DOCX 15 kb) [file 12883_2019_1322_MOESM2_ESM.docx]

Tab.2 Skeletal muscle ion channel genes with GC-rich (>70%) regions

| Name | Chromosome | Start | End | Length | %GC |
| --- | --- | --- | --- | --- | --- |
| ALG13 | chrX | 110924849 | 110925089 | 241 | 70.12% |
| KCNA2 | chr1 | 111149769 | 111149949 | 181 | 76.24% |
| KCNE3 | chr11 | 74178428 | 74178788 | 361 | 73.13% |

Tab.2 Skeletal muscle ion channel genes with GC-rich (>70%) regions.
